# The fire regimes of the Cerrado and their changes through time

**DOI:** 10.1098/rstb.2023.0460

**Published:** 2025-04-17

**Authors:** Carlota Segura-Garcia, Ane Alencar, Vera L. S. Arruda, David Bauman, Wallace Silva, Dhemerson E. Conciani, Imma Oliveras Menor

**Affiliations:** ^1^ Environmental Change Institute, School of Geography and the Environment, University of Oxford, Oxford, UK; ^2^ IPAM, Brasilia, Brazil; ^3^ AMAP, Univ Montpellier, IRD, CIRAD, CNRS, INRAE, Montpellier, France

**Keywords:** fire regimes, open ecosystems, disturbance ecology, land use effects, climate change

## Abstract

The Brazilian Cerrado is a heterogeneous region of open ecosystems adapted to fire intermingled with patches of woody growth-forms, with high levels of biodiversity and endemism. In recent decades, land conversion and human activities have proliferated across the Cerrado, losing about half of its original area. These changes, coupled with climate change, are altering its fire regimes with uncertain, but possibly adverse, consequences for Cerrado ecosystems. Here, we used burned area data to characterize the fire regimes of each cell on a 30 km grid over the Cerrado, and used a spatially constrained hierarchical clustering approach to delineate the regions with different fire regimes in four consecutive 9-year periods between 1985 and 2020. Comparing the periods 1985–1993 and 2012–2020, we found substantial changes in the number and shape of the fire regime regions, and in their fire characteristics. The main factor differentiating these regions was their level of fire activity: some showed large, numerous and frequent fires, while others showed small, few and infrequent fires. We also identified a region in the north with a later peak of the fire season, characterized by small but numerous fires. Finally, we found that while the fire activity of the southern areas of the Cerrado substantially decreased, fire activity levels in the centre and north increased or remained high over time.

This article is part of the theme issue ‘Novel fire regimes under climate changes and human influences: impacts, ecosystem responses and feedbacks’.

## 1. Introduction

Across the world, areas with different types of vegetation, climatic ranges and socio-economic characteristics present different patterns of fire occurrence, referred to as fire regimes [1–3]. The relationships between characteristics of fire like size, frequency, intensity or seasonality and the factors that affect them are complex, often forming feedbacks by which one factor influences the others and vice versa [4]. Further, the effects of different drivers of fire may become apparent only at certain scales—landscape, regional or global [3,5]. Fire regimes are a useful framework to study how the characteristics of fire vary geographically in relation to these factors [3], understand how fire may contribute to shaping different landscapes [6,7], weigh its contribution to carbon emissions and other biogeochemical cycles [8], and foresee how fire may respond to changes in the climate. At regional to local scales, characterizing fire regimes can also contribute towards the conceptualization of fire management strategies and conservation plans that are adequate to specific contexts.

Certain open ecosystems, like savannahs and grasslands, are adapted to fire as their vegetation has co-evolved with this phenomenon over millions of years [9,10]. Therefore, given a certain vegetation composition, it is argued that there exists an underlying fire regime the vegetation is specifically adapted to [9]. However, biomes of open ecosystems are usually very heterogeneous in their vegetation composition, topographic features and climatic characteristics and, as a consequence, can present different fire regimes across their area [2]. Furthermore, the expansion of modern human land use activities and anthropic climate change are rapidly altering the fire regimes of these regions [11]. Indeed, in tropical savannahs, humans shape fire regimes through landscape fragmentation [12], shifting the fire season [11,13] by igniting fires for agricultural and land conversion purposes [14,15], or actively preventing or suppressing fires—which may lead to more infrequent but larger and more intense fires as a result of fuel accumulation [16,17]. Regarding climate, even though there is established evidence that climate change is altering savannahs’ fire regimes [18], and that it will continue to do so [19,20], the specific long-term effects are still uncertain. While climate change is associated with surges in the number, size and intensity of fires in relation to increasing temperatures and the frequency of climate anomalies [21], or the lengthening of the dry season [18]; in the long run, the effects on fire regimes will also depend on its effects on vegetation productivity during the growing season, which ultimately influences the amount of available fuel [5,22]. In turn, the alteration of savannahs’ fire regimes as a result of the expansion of human activities and climate change may further couple with these two processes to affect the vegetation of these open ecosystems [23], as well as having broader effects on the planet’s carbon cycle [24].

The Brazilian Cerrado is a clear example of a large and heterogeneous mosaic of open ecosystems where both its landscape and fire regimes are undergoing rapid and profound changes in their configuration [25,26]. Indeed, despite its high levels of endemism and rich biodiversity [14,27], around 50% of the Cerrado’s 2 million km${,}^{2}$ have been converted to agricultural land uses in recent decades [28] roughly expanding from the south towards the Matopiba region in the north, the Cerrado’s current agricultural frontier [29]. Simultaneously, this region has also become warmer and drier [30] under climate change. Apart from its heterogeneous vegetation, the Cerrado presents a diverse topography, climate and soil composition, as well as varying levels of landscape fragmentation and different anthropic land uses, giving rise to different ecoregions [31]. All of these elements influence its fire regimes [22,32,33]. Indeed, studies across the Cerrado and its ecoregions have observed a great heterogeneity in the patterns of fire, with overall low burned area levels in the south of the Cerrado and higher levels in the centre of this region [25,26], as well as heterogeneous changes in burned area through time. Decreases in burned area are generally associated with increasing landscape fragmentation as a consequence of agricultural expansion, while increases in burned area are related to both climate change and the growing presence of human activities in the least converted parts of the Cerrado [25,34,35], as humans cause ignitions in relation to agricultural practices, accidents, fire management or in the conversion of certain vegetation forms [15,36]. However, a clear delineation of the different regions of the Cerrado presenting different fire regimes and how these may have changed over time is still lacking.

In this study, we analysed the fire regimes of the Brazilian Cerrado in the period 1985−2020. First, we aimed to identify the different regions of the Cerrado presenting distinct characteristics of fire in four 9 year periods between 1985 and 2020, with a particular focus on the first (1985−1993) and last (2012−2020) periods. For this purpose, we combined monthly burned area data with 30 m spatial resolution with advanced spatially constrained clustering methods to delineate the regions of the Cerrado with different fire regimes based on a 30 km grid. Then, we studied the characteristics of these regions in terms of various factors related to the presence of human activities in the landscape and in terms of various climatic variables that may explain the differences in the fire regimes of the various regions identified. Finally, we analysed how these regions have changed over time, both geographically and in terms of their fire characteristics, with the ultimate aim of understanding how the rapid changes the Cerrado has experienced over the past four decades have shaped its fire regimes.

## 2. Methods

### (a) Study area

The Cerrado is a heterogeneous Brazilian ecological region formed by a range of vegetation forms [37], predominantly savannahs and grasslands. The Cerrado occupies an area over 2 million km${,}^{2}$ spanning 11 Brazilian states and presents varying levels of anthropic land uses [28]. In the 1970s, large-scale agricultural expansion rapidly converted the Cerrado to anthropic land uses [38,39], affecting around 50% of its native vegetation area [28].

The Cerrado presents a latitudinal gradient of temperatures increasing towards the Equator, ranging from around 18${,}^{\text{o}}$C to 28${,}^{\text{o}}$C [40]. The climate of this biome is strongly seasonal, with a wet and a dry season (roughly May to October). In addition, there is a gradient of precipitation increasing from east to west, varying between 800 mm and over 1800 mm [40].

To characterize the fire regimes of these savannahs, we used a 30 × 30 km grid laid over the Cerrado and divided the study period in four 9 year periods: 1985−1993, 1994−2002, 2003−2011 and 2012−2020. The size of the cells was chosen to have enough spatial resolution, while being large enough to simultaneously limit the number of fire polygons larger than one cell (see § 2b(i)), as well as minimizing the number of cells with little to no fire events in a period.

### (b) Data sources and processing

#### (i) Burned area data and fire polygons

We used burned area data from MapBiomas Fogo (Collection 2.0, [41]), a publicly available dataset covering the period 1985−2022 based on Landsat images (30 m spatial resolution with a 16 day interval) and created using machine learning algorithms in the Google Earth Engine platform [42]. This dataset provides annual maps of burned area where pixels identified as burned are labelled with their mapping month. We downloaded the data using the MapBiomas toolkit for the aforementioned Google Earth Engine platform.

We processed this raster data using a custom algorithm to create fire polygons in vector format so that we could have an estimation of the size of the different fire events. Considering the limitations of Landsat data regarding the temporal resolution, as well as those possibly introduced by the mapping methodology, we mapped together as part of the same fire event (i.e. fire polygon) those spatially contiguous pixels belonging to consecutive months, as well as those polygons separated by up to 150 m from border to border. The reason was threefold: first, two physically separated fires occurring close to one another in space and time may be part of the same fire event [43]; second, Landsat’s spectral resolution and the mapping algorithm may fail to detect certain burned pixels within a fire event; and third, when simply mapping the polygons without this last condition, we found an excess number of small fires compared to the expected heavy-tail fire size distribution [44]. Finally, we kept only those fire polygons larger than 3 ha.

We quantified the fire regimes of a certain 30 km cell in a specific 9 year period by focusing our analyses on six fire variables: (i) *number of fires*, the number of polygons normalized to flammable area (see §2b(ii) ); (ii) the *50th quantile* and (iii) *99th quantile* of the polygons’ size distribution; (iv) *burned area*, the sum of burned pixels in the period normalized to flammable area; (v) *fire frequency* as the 99th quantile of the smoothed empirical cumulative distribution function (sECDF) of the number of times each cell’s pixels burned in the period (between 0 and 9); and (vi) *fire seasonality*, as the peak of the fire season using the median value of the sECDF of monthly burned area in a fire-year. We established the fire-year for each cell as the 12 month period centred at the month with highest burned area. This consideration was important given the latitudinal gradient observed in the peak of the fire season. Henceforth, we will refer to the fire regime of a grid cell or region in a period as the combination of these six quantities.

**Figure 1.**
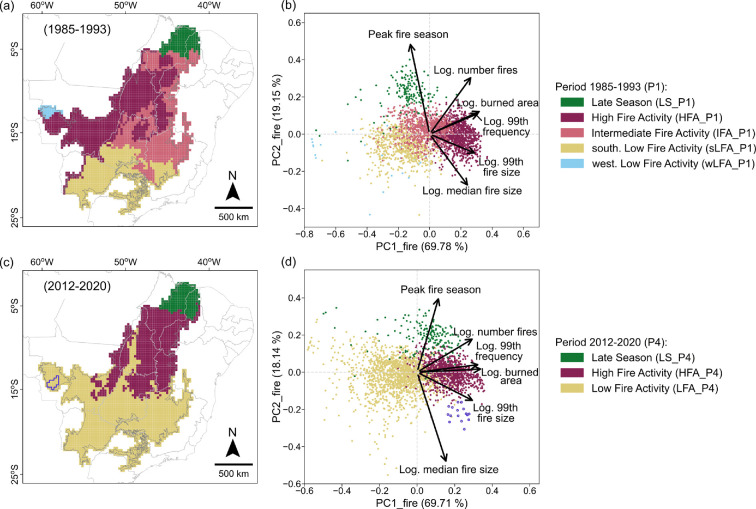
Fire regime regions obtained for (a) the first period (1985−1993) and (c) the last period (2012−2020) corresponding to the different clusters identified by the spatially constrained clustering algorithm performed on a 30 km grid over the Cerrado. Plots (b) and (d) present, for each period respectively, the first two axes (PC1_fire and PC2_fire) resulting from the principal component analysis performed on the six fire variables (PCA_fire): the median and 99th percentile fire sizes, the burned area and the number of fires, the 99th percentile of the fire frequency distribution and the median (peak) of the fire season. Each point in the PCAs represents a 30 km grid cell in the period coloured depending on the fire regime region it was classified into. Overall, in both periods, the PC1_fire captures a gradient of fire activity (see §3) and the fire regime regions appear ordered along this axis. In the first period, five different fire regime regions were identified: one in the north characterized by small numerous fires and a later peak of the fire season (LS_P1), a region in the centre-west with comparatively higher fire activity (HFA_P1), another region in the centre-east with intermediate fire activity (IFA_P1) and two regions with lower fire activity, one in the south (sLFA_P1) and another one in the western-most tip of the Cerrado (wLFA_P1). In the fourth and last period, three regions were identified: one in the north presenting a later peak of the fire season (LS_P4), one in the centre with higher fire activity (HFA_P4) and another one in the south with comparatively lower fire activity (LFA_P4). Note that even though the same colour scheme was used in both periods to denote analogous fire regime regions, neither the geographical limits nor the overall fire characteristics of the equally coloured fire regime regions between periods are necessarily the same.

Finally, a few grid cells in each 9 year period presented no burned area, indicating absence of fire. However, the clustering algorithm requires each cell to have a value for each fire variable. Therefore, we assigned these cells the minimum fire size (3 ha) both for the median and 99th percentile fire size and for burned area, and a frequency and number of fires of 1. We used the average peak of the fire season from the immediate eight neighbouring cells. There were few cells with no fire occurrences, and these were generally spread out across the south of the Cerrado, with the exception of a small group of contiguous cells presenting no fires in the western-most tip of the Cerrado in the period (1985−1993), which were classified as part of cluster wLFA_P1 (see §3 and figure 1).

Both the creation of fire polygons and the quantification of the six different fire variables in each 30 km grid cell and period were carried out in Python (Python Software Foundation, https://www.python.org/), using the packages GeoPandas [45], Pandas [46] and Rasterio [47], as well as the QGIS [48] Python Application Programming Interface (API).

#### (ii) Land use area data and fragmentation indices

We used annual maps of land use and land-cover from the MapBiomas project (Collection 8.0, [49]). For each grid cell in the first (1985−1993) and last (20120−2020) periods, we calculated the average percentage of anthropic area (comprising ‘urban area’, ‘mining’ and all land use categories under ‘farming’), ‘forest formation’, ‘savanna formation’, ‘grassland’ and ‘wetland’. Additionally, we calculated the change in anthropic area within each period as the difference in anthropic area between the last and the first year in the period. Finally, we calculated the average flammable area as the sum of all those categories describing landscapes with enough vegetation to sustain a fire (i.e. all categories except ‘hypersaline tidal flats’, ‘rocky outcrops’, ‘non vegetated area’ and ‘water’). We downloaded the land use and land-cover data using the MapBiomas toolkit for Google Earth Engine, and processed it using various Python packages (Python Software Foundation, https://www.python.org/).

We also calculated different types of landscape fragmentation indices using the Python package ‘PyLandStats’ [50]. However, these indices were not used in the final analysis after inspection of collinearity and variable importance using random forests algorithm (see below).

#### (iii) Climate reanalysis data

For each 30 km grid cell in the first (1985−1993) and last (2012−2020) periods, we calculated the average annual and dry season temperature and precipitation, and the peak of the dry season. We used the 2 m temperature variable from ERA5-Land Monthly Averaged climate reanalysis data [51] (9 km spatial resolution) from the European Centre for Medium-range Weather Forecasts (ECMWF). For precipitation, we used the 5 day precipitation accumulation in millimetres from the Climate Hazards Group InfraRed Precipitation with Station data [52] (CHIRPS, version 2.0), a quasi-global rainfall dataset with 0.05${,}^{\text{o}}$ spatial resolution. We chose the ERA5-Land dataset for temperature because the ECMWF data products have been identified as the most reliable air temperature datasets over tropical regions [53]. We used CHIRPS v2.0 for precipitation, as it is considered the best data source for precipitation across the tropics [53]—along with the Tropical Rainfall Measuring Mission [54]. Finally, we used the potential evapotranspiration (PET) data provided by TerraClimate [55], a global dataset of climatic data with 0.5${,}^{\text{o}}$ spatial resolution.

First of all, we calculated the average value of each climatic variable—temperature, precipitation and PET—for each 30 km grid cell, year and month as the average of all pixels of the corresponding data products intersecting the cell in the corresponding year and month (weighting each pixel’s contribution to the average depending on the pixel’s area within the cell, using the R package Exactextractr [56]).

For each period and grid cell, we considered the dry season to be those months in which, on average, PET exceeded precipitation. We identified the peak of the dry season as the month with the most negative climatological water deficit (CWD) on average. We calculated the CWD based on Malhi *et al*. [57], as the cumulative difference between precipitation and PET (${\text{CWD}}_{m}={\text{CWD}}_{m-1}+{\text{Prec.}}_{m}-{\text{PET}}_{m}$, $\text{Max}({\text{CWD}}_{m})=0$, where m represents a certain month) in every 12 month cycle starting on the wettest month on average of each grid cell and period. Finally, we calculated the average monthly CWD for each period and grid cell, and identified the month with the most negative value.

Finally, for each 30 km grid cell and period, we calculated the average annual temperature as the average temperature across all months in the period, the dry season temperature as the average temperature across dry season months, the annual precipitation as the average total precipitation in a year, and the dry season precipitation as the average total precipitation across dry season months.

We downloaded the climatic data from the Google Earth Engine platform using the Geemap Python package [58] and Jupyter Notebook [59]. In this case, we processed the data in R [60], using the package Exactextractr [56].

#### (iv) Population and livestock density data

For each grid cell in the first (1985−1993) and last (2012−2020) periods, we calculated the median annual population density and livestock density. The density data were obtained from the Instituto Brasileiro de Geografia e Estatística [61,62] at municipality level. We resampled these two sets of data from the municipality level to the grid level using various Python packages.

### (c) Analysis

Here, we used a combination of ordination and clustering techniques, as well as linear regressions, to address our research questions. As described in more detail below, we first summarized the main axes of variation of the six fire variables (burned area, frequency, largest and median fire sizes, peak of the fire season and number of fires) using principal component analyses (PCA) to characterize the fire regimes at the grid cell level in each 9 year period. We then used a spatially constrained clustering algorithm to identify the fire regime regions, that is, the geographical areas formed by each cluster of grid cells with similar combinations of fire variables (similar fire regimes). Next, we focused on the first (1985−1993) and last (2012−2020) periods as we were mostly interested in understanding how the fire regime regions and their climatic and anthropic characteristics may have changed between 1985 and 2020. For each of these two periods, we used PCAs and redundancy analysis to investigate the characteristics of the different fire regime regions in terms of the climate and the presence of human activities, and their association with the characteristics of fire. Finally, we explored how these fire regime regions changed through time geographically and also how their characteristics of fire evolved using linear regression on time series data.

#### (i) Clustering algorithm

In preparation for the clustering algorithm, we calculated the fire regimes for each cell and 9 year period, transformed seeking normality by taking the natural logarithm of the median and 99th percentile fire size, burned area, number of fires and fire frequency (after adding a unit). Then, for each 9 year period, we performed a PCA on the correlation matrix of the set of standardized fire variables [63] (henceforth PCA_fire). We ran the clustering algorithm on the first three axes of each period’s PCA as they described more than 95% of the variation in the data.

To determine the different fire regime regions of the Cerrado, we used a hierarchical clustering algorithm with a spatial contiguity constraint [64] because we were interested in delineating spatially contiguous regions where fire regimes have similar features in a certain period, and investigate the environmental characteristics that may explain such geographical patterns. As a result of such constraint, areas with similar fire regimes but geographically disconnected would be identified as different clusters—i.e. different fire regime regions. We explored the fire regime characteristics of each cluster using the first two components of the PCA_fire (PC1_fire and PC2_fire; electronic supplementary material, table S1).

Because the algorithm we used is an unsupervised method [64]—i.e. the classification does not use any *a priori* knowledge on the clustering of the data—we explored a few metrics to identify an optimal clustering solution (i.e. the number of clusters) delineating compact and dissimilar clusters. We finally based the decision on the bayesian information criteria (BIC) (electronic supplementary material, figure S1), although also consulting the Dunn Index and a silhouette plot.

Finally, we repeated the clustering procedure on the first (1985−1993) and last (2012−2020) periods excluding the peak of the fire season. The objective was to understand the distinctiveness of certain areas in absence of this fire attribute (see §3 and the electronic supplementary material, figure S9).

We carried out this part of the analysis using R [60]. In particular, we used the package vegan [65] to run the PCA and the function constr.hclust() [64] distributed with the package adespatial [66] to run the clustering algorithm.

#### (ii) Environmental characteristics of the clusters

Focusing on the first (1985−1993) and last (2012−2020) periods, we analysed the fire regime regions in terms of two sets of explanatory variables related to the level of anthropic activities in the landscape (percentage of anthropic area, the change in anthropic area percentage within the period and the population and livestock densities), and to the climatic characteristics (average annual and dry season temperature, average total annual and dry season precipitation and the peak of the dry season) of each fire regime region. For both groups of variables, we first assessed collinearity between variables and performed transformations as appropriate seeking to improve normality (electronic supplementary material, figures S3 and S4 and table S8).

For each group of explanatory variables separately and together, we first ran a random forests algorithm [67] to assess the classification power of each set and evaluate the variables’ predictive importance. Then, we performed a PCA on each set of variables in each of the two periods to analyse the climatic and anthropic characteristics of each region (henceforth PCA_clim and PCA_anth). Finally, we analysed the relationship between each of the first components of the anthropic (PC1_ant) and climatic (PC1_clim) variables and the PC1_fire. These post-hoc assessments of PC1 components and environmental variables were further tested using constrained ordinations—redundancy analyses (RDA), a method that allows us to use multiple explanatory variables to model simultaneously a set of response variables [63]—to test for the statistical significance and explanatory power of the environmental variables sets to explain the variation in the multivariate space of fire variables.

To run the random forests algorithm, we used the R package randomForest [68]. For the various PCAs and RDAs, we again used the package vegan [65].

#### (iii) Temporal changes of the fire regimes

We studied temporal changes in each of the six fire variables and the PC1_fire. To make the periods comparable, we projected the PC1_fire of each 9 year period on the PC1_fire of the first period. Then, we calculated the temporal changes of PC1_fire and of each fire variable by fitting a linear regression model to each cell using either the time period (for PC1_fire and fire frequency) or the year (for the other fire variables) as the predictor. Hence, we fitted


(2.1) $$\begin{array}{r} \begin{array}{r} {\text{x}}_{f}=\alpha +\beta t, \end{array} \end{array}$$

where x${,}_{f}$ represents PC1_fire or each of the fire variables, *t* is the time variable, α represents the average value of the grid cell at the beginning of each time series and β is the rate of change of x${,}_{f}$ over time. We fitted the model using ordinary least squares in Python with the statsmodels library [69]. We used an 80% confidence interval (CI) to assess the statistical significance of the trends.

Finally, because the PC1_fire—capturing the largest variation in the data—had a meaning in terms of the characterization of the fire regimes of the various regions (see §3), we further compared the PC1_fire value of each cell in the first (1985−1993) and last (2012−2020) periods to investigate whether this PCA_fire score had increased or decreased over time.

## 3. Results

### (a) The fire regime regions of the Cerrado

Using the hierarchical clustering algorithm with a spatial contiguity constraint and based on the BIC criteria, we identified a different optimal number of fire regime regions (the different clusters of grid cells located in a certain area of the Cerrado) for each 9 year period: five regions for the first period (1985−1993), and three regions for the following three periods (1994−2002, 2003−2011, 2012−2020) (figure 1; electronic supplementary material, figure S8).

Focusing on the first (1985−1993) and last (2012−2020) periods, the PCA_fire in these two periods (figure 1b,d; electronic supplementary material, table S1) showed a similar composition of the first and second axes (PC1_fire and PC2_fire, respectively) in terms of fire variables, and a similar proportion of the variance described (88.9% and 87.9% in the first and last periods, respectively). In both periods, burned area, fire frequency, largest fire size and the number of fires contributed the most, and in the same direction, to PC1_fire. Regarding PC2_fire, it was the peak of the fire season and the number of fires in one direction, and the median fire size in the opposite direction, that contributed the most. Further, even though the contribution of the 99th percentile fire size to PC2_fire was weaker than the three aforementioned variables, it did so in the same direction of the median fire sizes, and the opposite direction to the number of fires. This opposite direction between the size and the number of fires indicates a potential trade-off between these two variables of fires, even though we still observed some cells with simultaneously large and rather numerous fires.

In both periods, the clusters appeared ordered along PC1_fire indicating that, overall, cells with high levels of burned area, also had high levels of fire frequency, fire size or number of fires, and vice versa for cells with low and intermediate values (figure 1). That is, cells with similar overall low, intermediate or high levels of the various variables were generally classified as part of the same fire regime region. Therefore, the PC1_fire captured a gradient of increasing fire activity, and the level of fire activity (PC1_fire) was the main factor differentiating the different clusters.

In the first period (1985−1993), the clustering algorithm delineated a fire regime region whose grid cells presented the highest fire activity levels (henceforth HFA_P1) overall when compared to the other four regions. This HFA_P1 cluster was the largest region delineated in the first period (occupying 38% of the Cerrado), and it was located in the west of the Cerrado spanning a large latitudinal range. East of this region, the algorithm identified a fire regime region that also covered a large latitudinal variation and presented intermediate values for all fire characteristics (intermediate fire activity, IFA_P1). The algorithm identified two clusters with low fire activity when compared to the other fire regime regions, one located in the southern-most part of the Cerrado (sLFA_P1) and another located in the western-most tip of the Cerrado (wLFA_P1)—the smallest region identified in the first period. Finally, the last fire regime region delineated was located in the northern-most part of the Cerrado, standing out for presenting small but numerous fires and, particularly, a later peak of the fire season—the month when most fires occur—compared to the other regions (late season region, LS_P1). Particularly, most fires in the LS_P1 fire regime region took place between October and November, while the peak of the fire season for the other clusters took place between August and September (electronic supplementary material, figure S2f).

In the fourth and last period (2012−2020), the algorithm again identified a fire regime region with overall higher fire activity (HFA_P4) when compared to the other fire regime regions, located in the central part of the Cerrado. In addition, the algorithm identified a region in the south with overall lower levels of fire activity (LFA_P4), except for a set of cells showing some of the largest fires (highlighted in blue in figure 1c,d). In this last period, however, this low fire activity region was much larger than in the first period, spanning 59.5% of the Cerrado. Finally, there was only one fire regime region that persisted from the first period, presenting again, overall high number of fires and a later peak of the fire season (late season region, LS_P4). In this last period, however, the size of the largest fires observed in LS_P4 were larger than those in LS_P1, and comparable in size to HFA_P4 (figure 1d). Again, while the peak of the fire season of grid cells in LS_P4 took place in October and November; the peak of the fire season for the other two regions was between August and September (electronic supplementary material, figure S9f).

Finally, we also explored the relative importance of the peak of the fire season when delineating the fire regime regions by excluding this variable from the clustering. Even when not including the peak of the fire season, the fire regime region in the north was still delineated in the first period (electronic supplementary material, figure S9a,b), while in the last period, it was classified as part of the high fire activity region but still presented a distinctive combination of fire characteristics in comparison to other grid cells in the same fire regime region (electronic supplementary material, figure S9c,d).

### (b) Environmental characteristics of the fire regime regions

**Figure 2.**
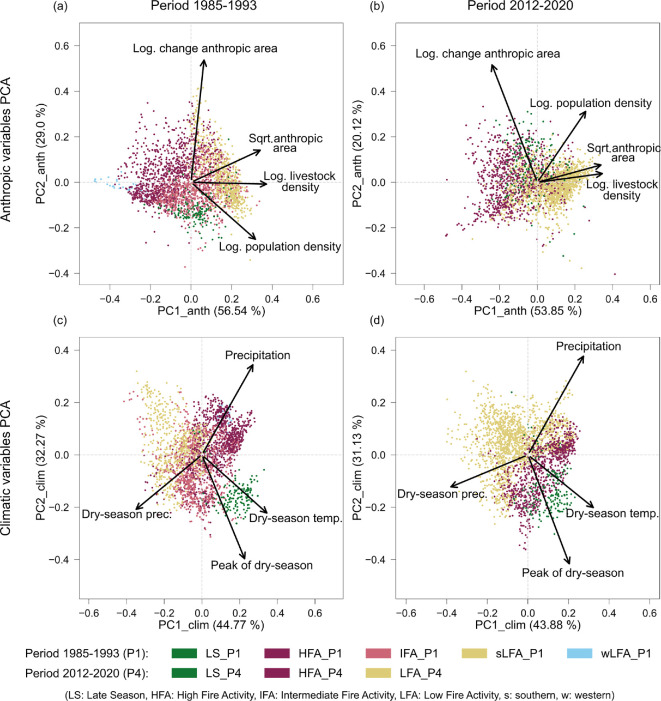
The two major axes of variation obtained from the PCA performed on each set of explanatory variables, anthropic and climatic variables. The anthropic variables (PCA_anth) used in the first (a) and last periods (b) were anthropic area percentage, livestock density, population density and change in anthropic area percentage within the period. The climatic variables (PCA_clim) used in the first (c) and last (d) periods were the total annual precipitation, total precipitation in the dry season, average temperature in the dry season, and peak of the dry season. Points represent a 30 km grid cell in the corresponding period coloured according to the fire regime region they were classified into (see figure 1). Note that even though the same colour scheme was used in both periods to denote analogous fire regime regions, neither the geographical limits nor the overall fire, anthropic or climatic characteristics of the equally coloured fire regime regions are necessarily the same.

Exploring the climatic characteristics and the level of human activities of the different regions, both PCA_clim and PCA_anth revealed that the fire regime regions obtained independently through the clustering algorithm appeared well separated by each set of explanatory variables in each period (figure 2), indicating that the variation in these variables shared geographical structure with that of the fire variables. Further, we evaluated the ability of the explanatory variables to classify the fire clusters running a random forests algorithm. The classification accuracy was very high in both periods for both the sets of anthropic (81.7 [78.1 84.9]% and 82.3 [79.3, 85.9]%, both with 95% , CI) and climatic (82.3 [78.7, 85.4]% and 90.0 [87.1, 92.4]%, 95% CI) variables (electronic supplementary material, table S2). This accuracy was even higher when using both sets of variables together (88.7 [85.9, 91.2]% and 94.0 [91.8 and 95.8]% in each period, respectively, 95% CI).

Regarding the PCA_anth, in both periods PC1_anth was contributed primarily by the percentage of anthropic area, and the livestock and human population densities, all pointing in the same direction (figure 2a,b; electronic supplementary material, table S4). Therefore, this axis seemed to capture the variation in the level of anthropic presence and activities in the landscape. Moreover, the clusters appeared aligned along this gradient. In the first period, region sLFA_P1 in the south presented the highest levels of anthropic activity, followed by regions IFA_P1 in the centre-east, LS_P1 in the north, and part of cluster HFA_P1 in the centre-west; while region wLFA_P1 in the western-most tip of the Cerrado and most of region HFA_P1 presented the lowest levels of anthropic presence (figure 2a). In the last period, the region LFA_P4 in the south had the highest levels of anthropic presence, followed by regions LS_P4 in the north and HFA_P4 in the centre, in opposite order to the gradient of fire activity of these regions (figure 2b). In both periods, the change in anthropic area within the period was the variable that contributed the most to PC2_anth.

Considering the PCA_clim, both in the first and last periods, the dry season temperature and mean annual precipitation in one direction, and the dry season precipitation in the opposite, contributed the most to PC1_clim, while the peak of the dry season contributed the most to PC2_clim (figure 2c,d; electronic supplementary material, table S6). In this case, PC1_clim seemed to capture a gradient of availability of fuel and suitability of conditions to burn, that is, the flammability: hotter and drier climate in the dry season, when most of the fires occur [20]; and amount of annual precipitation, which contributes to vegetation growth [5,22]. In this case, however, the clusters aligned along the direction of dry season temperature—hence following the latitudinal gradient—and less perfectly along the flammability gradient (PC1_clim). Finally, in both periods, the northern regions LS_P1 and LS_P4 presented a later peak of the dry season than the other regions.

**Figure 3.**
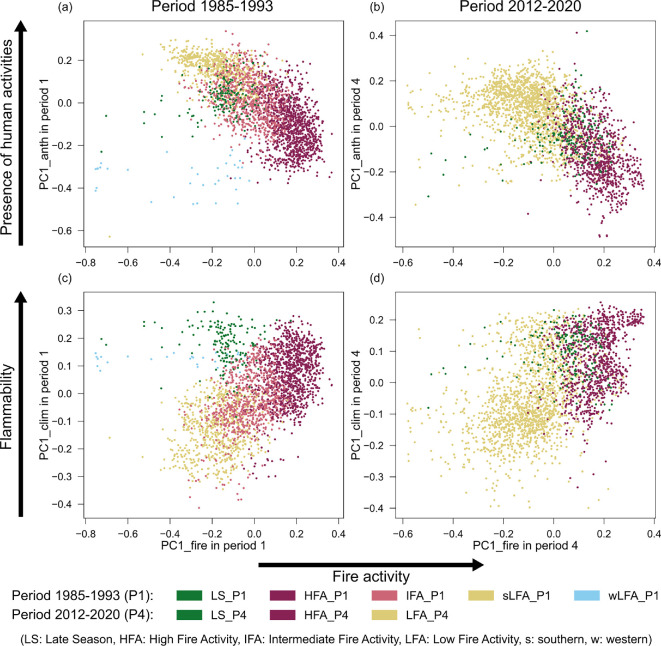
Relationship between the first component of the PCA performed on the anthropic variables (PC1_anth), (a) and (b) in the first and last periods, respectively, or the climatic variables (PC1_clim), (c) and (d) in each period, respectively, and the first component of the PCA performed on the fire variables (PC1_fire). PC1_fire in each period represents a gradient of increase in burned area, frequency and the size and number of fires (figure 1a; electronic supplementary material, table S1), capturing a gradient of fire activity. PC1_anth in both periods represents a gradient of increase in anthropic area, population and livestock density (figure 2a,b, electronic supplementary material, table S4), that is, an increase in the presence of human activities and land use area in the landscape. Finally, PC1_clim represents a gradient of flammability conditions, with an increase in dry season temperature and in annual precipitation (contributing to vegetation growth [5,22]), and a decrease in dry season precipitation, when most fires occur [20] (figure 2c,d; electronic supplementary material, table S6). Points represent a 30 km grid cell in the corresponding period coloured according to the fire regime region they were classified into (see figure 1). Note that even though the same colour scheme was used in both periods to denote analogous fire regime regions, neither the geographical limits, nor the fire, anthropic or climatic characteristics of the equally coloured fire regime regions are necessarily the same.

Lastly, since the PC1 axes represented environmental gradients, we looked at their relationship with the fire activity gradient (figure 3). On the one hand, PC1_anth and PC1_fire were inversely related: as the presence of humans and their activities in the landscape decreased (lower PC1_anth), fire activity increased (higher PC1_fire) for all clusters in both periods (figure 3a,b). The only exceptions to this relationship were region wLFA_P1 and some cells in the northern region LS_P1 that presented a lower fire activity than other cells with the same level of human presence. On the other hand, PC1_clim and PC1_fire were directly related: as the flammability increased (higher PC1_clim), fire activity also increased in both periods (figure 3c,d). Again, most cells in regions wLFA_P1 and LS_P1 presented lower fire activity than those cells in region HFA_P1 with similar flammability scores.

These negative and positive correlations with fire activity of human presence and flammability, respectively, were further confirmed when modelling the correlations of each set of explanatory variables with the fire variables using an RDA approach (*r*${,}_{\text{adj}}^{2}$ = 0.316, 0.305 for anthropic and *r*${,}_{\text{adj}}^{2}$ = 0.242, 0.305 for climatic variables in each period, respectively, and *p*‐value < 0.001 for all variables and cases; electronic supplementary material, figure S6 and tables S5 and S7). These models further revealed that precipitation was only weakly correlated with the fire variables, and that there was a strong correlation between the peak of the dry season and that of the fire season (electronic supplementary material, figures S6c,d, and table S7).

### (c) Changes of fire regime regions over time

**Figure 4.**
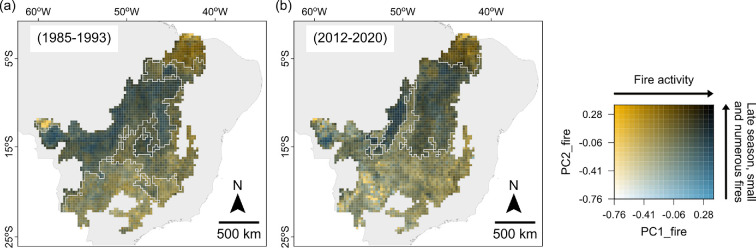
Bivariate maps showing the PCA_fire coordinates of each 30 km grid cell in the first and last periods projected on the PCA_fire-space obtained for the first period PCA_fire. Map (a) shows the PCA_fire scores directly obtained from the fire variables in the first period (1985−1993). Map (b) shows the projection of the fire variables of the last period (2012−2020) on the PCA_fire-space of the first period. In this manner, the fire regimes (six-dimensional) are summarized into two dimensions, and the values of the grid cells in each period can be compared. PC1_fire (*x*-axis of the colour map) represents an increase in fire activity (larger burned area, frequency and larger fire sizes) from white to blue, while PC2_fire (*y*-axis) represents the direction of a later fire season, an increase in the number of fires and a decrease in median fire size from white to yellow.

Between the first (1985−1993) and last (2012−2020) periods, there was a reduction in the number and a change in the shape of the fire regime regions (figures 1 and 4). Moreover, the last period clusters were formed by grid cells coming from a mix of different first-period regions (figure 4).

**Figure 5.**
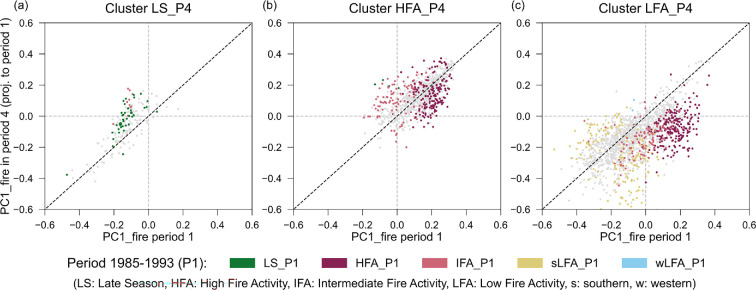
Relationship between PC1_fire obtained in the first period, 1985−1993 (*x*-axis), and the projection of PC1_fire obtained for the last period, 2012−2020, projected on the PCA_fire of the first period (*y*-axis). (a) Subset of 30 km grid cells classified into the northern fire regime region in the last period with small numerous fires and a later peak of the fire season, LS_P4; (b) subset of grid cells classified into the high fire activity region of the last period, in the centre of the Cerrado, HFA_P4; and (c) cells classified into the lower fire activity region of the last period, in the south, LFA_P4. Points represent 30 km grid cells and are coloured depending on the fire regime region they were classified into in the first period if they present clear trends in time in PC1_fire (with 80% confidence interval), or coloured in grey if they do not present clear trends in time. The dashed black lines represent the one-on-one correspondence between PC1_fire in the first period and the projection of PC1_fire in the last. Thus, points above the dashed black line represent an increase in fire activity over time, while data points below the line represent a decrease. Dashed grey lines represent the 0 values of PC1_fire and are included for reference.

**Figure 6.**
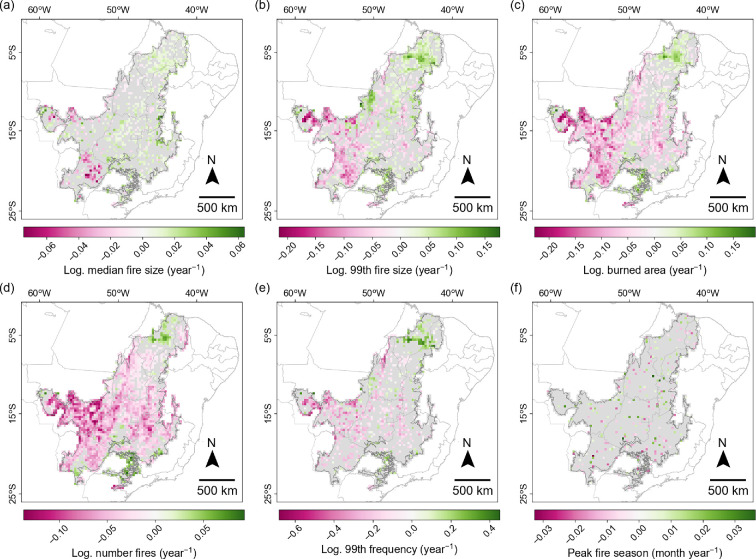
Trends in time (slope coefficients) obtained by fitting a linear regression (using ordinary least squares) to the time series of each of the six fire variables, for each 30 km grid cell over the Cerrado. Plots show the median fire size (a), the 99th percentile fire size (b), burned area (c), the number of fires (d) and the median fire season (f). The time series for these five variables comprised the annual values for each year in the period 1985−2020. The time series for the 99th percentile of the fire frequency distribution (e) comprised the frequency value in each of the four periods, since this value expresses the largest number of times the pixels in a cell burned in 9 years. Pink indicates decreases in the fire variable over time, green indicates increases and grey indicates no clear trends in time with 80% confidence interval.

Apart from these geographical changes, the fire characteristics of the individual grid cells also changed over time. In particular, there were increases and decreases in fire activity heterogeneously distributed across the Cerrado (the PC1_fire score; figure 5; electronic supplementary material, figure S7). Thus, even though the fire regime regions were organized along a gradient of fire activity in both periods, the levels of high fire activity of region HFA_P1 and low levels of regions sLFA_P1 and wLFA_P1 in the first period, did not perfectly correspond to the levels of high fire activity of region HFA_P4 and low fire activity of region LFA_P4 in the last period (figure 5b,c). Furthermore, the last period clusters were not the result of unidirectional changes, but rather the result of changes in different directions of each of the different fire variables (figure 6).

Clusters LS_P1 and LS_P4 in the north comprised the only region whose geographical shape and location did not substantially change between periods, and maintained its unique attribute of a later fire season peak. Regarding fire activity, however, 29% of the LS_P4 area experienced an increase in fire activity over the study period (figure 5a). On the other hand, region HFA_P4 in the centre of the Cerrado—with the highest levels of fire activity in the last period—was composed of cells showing increases (14.3% of its area) and decreases (15.9%) in fire activity over time (figure 5b). However, these decreases were limited and remained mostly within the range of activity of cluster HFA_P1 (around a minimum value of 0 in the PC1_fire, figure 5b). Regarding region LFA_P4 in the south—the cluster with lowest fire activity in the last period—27.1% of its cells experienced decreases in fire activity (figure 5c) over the study period. Most of its cells transitioned from clusters HFA_P1 and IFA_P1—initially with high and intermediate fire activity—experiencing mild to strong decreases in fire activity (47.9% and 22.8% of each cluster, respectively), while 16.9% of the cells transitioning from region sLFA_P1—already with low levels of fire activity—experienced further reductions.

## 4. Discussion

In this study, we identified the regions of the Cerrado presenting different fire regimes, the fire regime regions, using a hierarchical clustering algorithm with a spatial contiguity constraint in each of four 9 year periods between 1985 and 2020. We identified five fire regime regions in the first period, 1985–1993, and three fire regime regions in the last period, 2012–2020, and in the two periods in-between (figure 1; electronic supplementary material, figure S8). These regions reflect the spatial patterns of fire across the Cerrado and allowed us to explore how its fire regimes have changed over time. We found considerable differences in the geographical shape of the fire regime regions between the first and last periods, as well as in the fire characteristics of the individual 30 km grid cells that form the different regions (figures 4 and 5).

In each period, the fires regime regions of the Cerrado were primarily differentiated by their level of fire activity, from low fire activity—that is, with overall infrequent, small and non-numerous fires—to high fire activity—with overall frequent, large and numerous fires (figure 1b,c). This gradient of fire activity was captured by the first component of the PCA performed on the six fire variables (PCA_fire), along which the fire regime regions appear ordered (figure 1). Therefore, the primary feature differentiating the fire regimes of the Cerrado is their overall level of fire activity, prevailing over other differences in fire characteristics, in agreement with previous studies characterizing the fire regimes of the Cerrado [25]. Still, we observed a certain trade-off between the size and the number of fires (figure 1b,d), indicating that the grid cells with the largest fires did not experience the highest numbers of fires, and vice versa. The trade-off between these two variables of fire is related to the fact that when a large fire occurs, it consumes a large portion of the landscape’s fuel bed, thus inevitably hampering the occurrence of other fires in the same period and limiting the overall number of fires, as reported in previous studies [70–73].

The Cerrado is very heterogeneous in terms of vegetation [14,37], topography and soil composition [31], climate [40], hydrology [74] and land use area [49]. Based on these factors, different ecoregions have been described in the Cerrado [31], and their patterns of fire have been subsequently quantified [25]. Because these factors affect the patterns of fire [75], we could expect some similarities between the fire regimes of these ecoregions [25] and the fire regime regions identified here. Even though neither the number nor the overall geographical shape of the ecoregions (electronic supplementary material, figure S10) corresponds to those of the fire regime regions identified (figure 1), we did find some similarities. For example, the ecoregions located in the centre of the Cerrado were found to present large burned area and fire sizes overall [25], in agreement with the high and intermediate fire activity regions identified here (figure 1). In particular, the Bananal ecoregion in the centre-west of the Cerrado (electronic supplementary material, figure S10; [31]) appears clearly delineated as part of the high fire activity region of the last period (HFA_P4), presenting some of the highest levels of fire activity (figure 4), in accordance with this ecoregion’s fire regimes [25,34]. Similarly, the ecoregion Chapadão do São Francisco (electronic supplementary material, figure S10; [31]), characterized with large burned areas and fire sizes [25], can also be recognized as part of the high fire activity region of the first period (HFA_P1).

We did find, however, a remarkable difference between our fire regime regions and the ecoregions located in the south-west of the Cerrado, comprising the states of Mato Grosso and Mato Grosso do Sul (electronic supplementary material, figure S10). The ecoregion covering this area, Paraná Guimarães (electronic supplementary material, figure S10; [31]), was classified as having intermediate levels of burned area and big fires in the period 2001−2019 [25]. However, we observed the lowest levels of fire activity in the period 2012−2020 precisely within this area (LFA_P4; figure 4). This difference in the level of fire activity could be related to the fact that the Paraná Guimarães ecoregion includes part of the high fire activity region (HFA_P4) identified here, which could be contributing to the higher burned area and larger fire sizes reported for the whole ecoregion [25]. This highlights the relevance of exploring patterns of fire at different scales, as it can reveal different processes and dynamics [5].

The fire regime regions we identified are overall large and somewhat heterogeneous in the fire characteristics of their grid cells (figure 4). For example, we observed a subregion of large fires located within the low fire activity region of the last period (LFA_P4; highlighted in blue in figure 1c,d). Hierarchical clustering algorithms with a spatial contiguity constraint generate solutions (i.e. classifications for different numbers of clusters) by grouping together grid cells and clusters consecutively only if they are spatially contiguous [63,64]. Here, we focused on the first optimal clustering classification of the data, which we found to discriminate generally large fire regime regions primarily in relation to their level of fire activity. However, there could be further statistically relevant clustering solutions delineating clusters at smaller scales [76], which could uncover further patterns of fire regimes—perhaps recovering some of the Cerrado’s ecoregions—opening an avenue for future studies of the Cerrado’s fire regimes.

In terms of the two major drivers of fire, humans and the climate [11,77,78], both the anthropic and the climatic variables have high classification accuracy of the fire characteristics (electronic supplementary material, table S2), as expected [75,79]. We found that the fire regime regions appear distributed along the first axis of the PCA performed on the anthropic variables (PC1_anth; figure 2a,b), which indicates a gradient of increasing presence of human activities. This anthropic presence is particularly associated with agriculture—land use area and livestock density—and, to a lesser extent, with population density (figure 2a). Importantly, we observe that as anthropic presence increases, fire activity decreases (figure 3), in agreement with previous studies relating the expansion of land use area and fragmentation in savannas with decreases in burned area [25,34,35,77,80,81].

Indeed, both land use area and livestock grazing are associated with smaller fires and reduced burned area as they create fuel discontinuities through landscape fragmentation [75,78] and diminishing fuel loads [82,83]. Population density, however, has a more intricate relationship with fire activity as humans can ignite fires in higher or smaller numbers [75,78], but they can also limit fire activity through fire suppression efforts [11,82]—a widespread practice in the Cerrado [73,84,85]. On the other hand, we observed the highest level of fire activity in the areas of the Cerrado with larger remnants of native vegetation (electronic supplementary material, figures S2 and S3), in agreement with previous studies [25,26,34,35]. These dynamics in the more intact areas of the Cerrado may result from a confluence of factors comprising human-started fires escaping from nearby agricultural lands into native vegetation [15] and the timing of ignitions in the dry season [15], when the fuel bed of native vegetation is more flammable and effectively more continuous [86].

The western fire regime region in the first period (wLFA_P1) is, however, an exception to the inverse relationship between the gradient of human presence and that of fire activity (figure 1a,b). This fire regime region simultaneously presents both the lowest fire activity levels and the lowest presence of human activities in this period (figures 1b and 2a). The low fire activity of this region could be the result of a vegetation composition closer to that of the Amazon tropical rainforest, where natural fires are extremely rare [12,87] owingto moister conditions and a closed canopy with scant surface fuels, particularly when human presence is low. The contrast in terms of human presence between this region and the other low fire activity region in the same period (sLFA_P1) also shows how areas with different landscape characteristics can present similar fire regimes [2].

The distribution of the fire regime regions on the PCA_clim seems to reflect their geographical location, as they appear ordered along the latitudinal gradient of temperature [40], with the northern regions (LS_P1 and LS_P4) presenting the hottest values, and the southern regions (sLFA_P1 and LFA_P4) presenting the lowest values (figure 2c,d). Perpendicularly, the fire regime regions appear organized along the direction of increase in annual precipitation from east to west [40]. For example, in the first period, the intermediate fire activity (IFA_P1) in the east presents lower annual precipitation than the high fire activity (HFA_P1) and the western low fire activity (wLFA_P1) regions. Still, the first axis of the PCA_clim captures a gradient of climatic flammability conditions (figure 2c,d), which is positively related with fire activity (figure 3c,d). That is, fire activity increases with drier and hotter conditions in the dry season as expected—when most fires occur—[20,88–90], as well as with greater annual precipitation, which contributes to productivity during the growing season [22] and fuel load [5]. However, we observed that, comparing cells with similar high climatic flammability values, some cells with high presence of anthropic activities in the last period (region LFA_P4) have lower fire activity than cells with low anthropic activity levels (region HFA_P4), suggesting an interaction between humans and climate. Indeed, it has been demonstrated that the effect of the climate on fire is subject to the level of anthropic presence in savannahs [35,70,81].

Only one fire regime region was consistently identified in each period (LS_P1 and LS_P4; figure 1; electronic supplementary material, figure S8) presenting a later peak of the fire season. In addition, this area presents small but numerous fires, which makes this region stand out even when excluding the peak of the fire season from the clustering analysis (electronic supplementary material, figure S9). That is, this region’s fire regimes are distinct from those of the other fire regime regions and not merely shifted in time. A number of factors may explain the particularities of this region’s fire regimes. First, this area comprises the Floresta de Cocais and Costeiro ecoregions (electronic supplementary material, figure S10; [31]), which is noteworthy for being an ecological transition region between tropical dry forest, rainforest and savannha [91] composed primarily of broadleaf tree species [92] and palm trees [31]. In fact, in early versions of the Cerrado’s geographical limits, this area was not fully included and was classified instead as either a mix of Amazon and Cerrado [93] or as dry broadleaf forest [94]. Second, this area also presents unique climatic characteristics as it is affected by the Intertropical Convergence Zone, which carries air moisture from the Atlantic towards the Amazon rainforest [40], and presents an overall later peak of the dry season (figure 2). Third, in terms of human presence, this area presents a number of municipalities with high densities of *quilombola* population [95], communities of people with African descent [95] who traditionally use fire to renew native pastures or as part of shifting cultivation practices, among other uses [83]. Finally, this area is found within the Matopiba region, considered the Cerrado’s current agricultural frontier and where land conversion has been more widespread in recent years [29,88,96].

We observed opposing trends over time in fire activity across the Cerrado (figure 5), in agreement with previous studies [25,35]. In the northern half of the Cerrado, comprising the Matopiba—encompassing regions LS_P4 and HFA_P4—we observed the largest concentration of increases in fire activity. The surges in fire activity of these regions could be the result of the use of fire in land conversion or as part of modern agricultural practices [14,15] alongside large native vegetation remnants with little fuel discontinuities, and in the context of climate change. On the other hand, the southern half of the Cerrado mostly experienced decreases in fire activity. Such reduction was particularly striking in the state of Mato Grosso (electronic supplementary material, figures S7 and S10), in agreement with previous studies [25,34], which has undergone extensive Cerrado vegetation fragmentation owing to conversion to pastures and croplands in the 1990s and early 2000s [28,39]. Further, the shape of the low fire activity region in the southern half of the Cerrado in the last period (LFA_P4) clearly reflects the geographical patterns of anthropic land uses [28], highlighting the depth of the footprint of land use area on the Cerrado’s fire regimes.

Finally, between 1985 and 2020, many aspects of the fire management policies across Brazil have changed. Indeed, at the beginning of the study period, the use of fire had been banned by successive laws since colonial times [23], further supported by misunderstandings about the ecology of fire-adapted ecosystems and the occurrence of destructive fires in iconic fire-sensitive ecosystems. In the late 1990s, however, there was a shift in the discussion as fire started to be recognized as a disturbance agent that could promote and sustain Cerrado biodiversity [84]. There was hence a push to (re)introduce fires into protected areas, which eventually translated into fire policy changes [23]. Indeed, the 2012 New Forest Code contemplated the use of fire in fire-adapted ecosystems for ecological and cultural purposes. Therefore, part of the increase in fire activity that we observe in certain areas of the Cerrado could be related to the use of prescribed fires as a management tool. However, prescribed burns are usually performed in the early dry season [71,97], while most Cerrado fires currently occur later in the dry season [34], indicating that other uses of fire may still prevail. In this sense, the recently approved Brazilian National Policy on Integrated Fire Management is a promising avenue for Cerrado conservation, as these practices are demonstrating to be an effective tool in Cerrado conservation and preventing more extreme fire behaviour [73,97].

## 5. Conclusion

Our findings indicate that the main factor differentiating Cerrado’s fire regime regions is the level of fire activity, which is inversely related to the extent of modern human presence in the landscape. On the one hand, we report that recent agricultural expansion and fragmentation have profound effects on the Cerrado’s fire regimes by primarily diminishing fire activity. Indeed, high livestock densities, modern mechanized agricultural practices and croplands such as soybean plantations where fire use is limited now occupy large areas of the Cerrado. On the other hand, climate change could be setting the stage for increased fire sizes and frequency in areas where most native vegetation remains. Therefore, both agricultural expansion and climate change could impact Cerrado’s vegetation and contribute to its homogenization and loss of biodiversity, highlighting the profound multi-faceted effects of large-scale Cerrado native vegetation loss. Future research efforts should be allocated to decipher the variation in fire patterns at finer spatial scales, as well as establishing in more detail the causal relationship between the different drivers and specific fire characteristics. This knowledge about the Cerrado’s fire regimes is essential to develop meaningful policies aimed at conserving this unique biome in the context of global change.

## Data Availability

The climate data used in this study are publicly available from ERA5-Land Monthly Averaged at [98] , the Climate Hazards Group InfraRed Precipitation with Station data version 2.0 at [99], and TerraClimate at [100]. The land use data are publicly available from MapBiomas collection 8.0 at [101]. The burned area data are publicly available from MapBiomas Fogo Collection 2.0 at [102]. Livestock density data for Brazil is available at https://sidra.ibge.gov.br/tabela/3939 and population density of Brazil https://www.ibge.gov.br/estatisticas/sociais/populacao/9103-estimativas-de-populacao.html. The processed data (including the custom fire polygons created from MapBiomas Fogo Collection 8.0) and custom code are available at https://doi.org/10.5281/zenodo.14025420. Supplementary material is available online [103].
